# Deliver on Time or Pay the Fine: Scheduling in Membrane Trafficking

**DOI:** 10.3390/ijms222111773

**Published:** 2021-10-29

**Authors:** Giampaolo Placidi, Carlo C. Campa

**Affiliations:** 1 Italian Institute for Genomic Medicine, c/o IRCCS, Str. Prov.le 142, km 3.95, 10060 Candiolo, Italy; placidi.borsisti@iigm.it; 2 Candiolo Cancer Institute, FPO-IRCCS, Str. Prov.le 142, km 3.95, 10060 Candiolo, Italy

**Keywords:** sorting, endosome, ESCRT, retromer, commander, lipid rafts, PtdIns(3)P, Rab5, Rab7, Rab11

## Abstract

Membrane trafficking is all about time. Automation in such a biological process is crucial to ensure management and delivery of cellular cargoes with spatiotemporal precision. Shared molecular regulators and differential engagement of trafficking components improve robustness of molecular sorting. Sequential recruitment of low affinity protein complexes ensures directionality of the process and, concomitantly, serves as a kinetic proofreading mechanism to discriminate cargoes from the whole endocytosed material. This strategy helps cells to minimize losses and operating errors in membrane trafficking, thereby matching the appealed deadline. Here, we summarize the molecular pathways of molecular sorting, focusing on their timing and efficacy. We also highlight experimental procedures and genetic approaches to robustly probe these pathways, in order to guide mechanistic studies at the interface between biochemistry and quantitative biology.

## 1. Introduction

The intracellular transport of molecules between membrane-bound compartments ensures the current distribution of both proteins and lipids in cells. The key advantage of membrane-based transport compared to the free diffusion of molecules in cytosol relies on: (i) protecting molecules from undesired biochemical reactions; (ii) improving accurate delivery of cargoes to defined organelles; and (iii) providing a sustained and controlled release of molecules from cellular compartments. While a number of critical barriers must be overcome to achieve specific molecular targeting, ultimately, in most cases, the ability of molecules to be delivered at the site of their action is governed by the timing that is required by cells to sort them.

The molecular sorting process is based on the grouping of molecules based on shared similar properties and arranging them in a sequence that is ordered by some criterion and dependent on both cell type and condition. In mammalian cells, sorting pathways are ubiquitous. Both protein and lipid machineries required for molecular sorting are present in all cell types. Therefore, it is not by chance that even when expressed in nonpolarized cells, both apical and basolateral proteins could be sorted into different cargo vesicles [1,2,3,4]. Nonetheless, cell-type-specific variations are present. Such dissimilarities conceal cellular schemes responsible for differential sorting, further complicating their analysis.

Cell-type-specific sorting patterns arise as a consequence of differential expression of key trafficking components, a transport process that strictly depends on cell physiological status [5,6,7,8,9]. The redirection of membrane cargoes in response to environmental cues it is a well-recognized feature of membrane sorting, as exemplified by the Na,K-ATPase, an enzyme that controls ion gradients across cellular membranes. Although this ion pump is considered a canonical basolateral protein, it localizes to the apical side of retinal pigment epithelium cells while other standard trafficking markers retain their characteristic distributions [10,11,12,13,14,15,16,17].

In addition, it is well recognized that certain receptors are trafficked based on ligand type and ligand concentration and, as demonstrated, for the epidermal growth factor receptor (EGFR). EGFR shifts between degradation and recycling in response to decreased affinity and concentration of ligands [18,19]. Despite this evidence, it is less understood whether the distribution of other commonly studied cargoes is subjected to control by either a certain ligand, cell type, or cell status.

Theoretically, when investigating membrane transport, it is crucial to understand if molecules being sorted are arranged in a defined temporal sequence. Determining the sequence of events and their kinetics at the sorting station can provide important information about: (i) the overall efficiency of transport; (ii) the cost of the process; and (iii) the number of cellular activities required to manage and deliver cargoes [20,21]. In parallel, identification of the spatial position of molecules during the sorting process could predict the final intracellular fate of cargoes [22]. However, reporting transport mechanisms without knowing such details is a common practice in cell biology, probably due to the technical difficulties inherent in investigating the sorting process. Therefore, the majority of studies add little to our knowledge of mechanisms that deal with the complex and repetitive processes required to sort molecules in cells.

## 2. Monitoring and Analysis of Molecular Sorting

There are many compartments in eukaryotic cells presenting the ability to sort molecules (e.g., Golgi apparatus, endoplasmic reticulum, and plasma membrane). Among them, the endolysosomal system is the most fascinating, based on its plasticity and intense activity. The endocytic system comprises a large number of membranes and tubules with different shapes and distinct molecular compositions. In particular, four types of membrane-bound structures characterize the endolysosomal system: early endosomes (EEs), late endosomes (LEs), recycling vesicles, and lysosomes. Each type is deputed to perform a specific task and is connected to the others by defined trafficking routes. The early endosome is the principal sorting apparatus of the endolysosomal system and, therefore, it is also referred to as the sorting endosome.

Early/sorting endosomes accept molecules from major endocytic routes (i.e., clathrin meditated, clathrin-independent/dynamin dependent, clathrin independent/ dynamin/independent, Macropinocytosis, phagocytosis, caveolin-based) through Rab5 and EEA1-dependent pathways [23]. From the early endosome, cargoes can either be recycled back to the cell’s surface or can remain with the vacuolar portion of the endosome and be processed for degradation. In addition, molecules entering the sorting endosome could also be directed toward lysosome, Golgi, and other autophagic compartments, thereby complicating the evaluation and prediction of a cargo’s fate.

**Figure 1:**
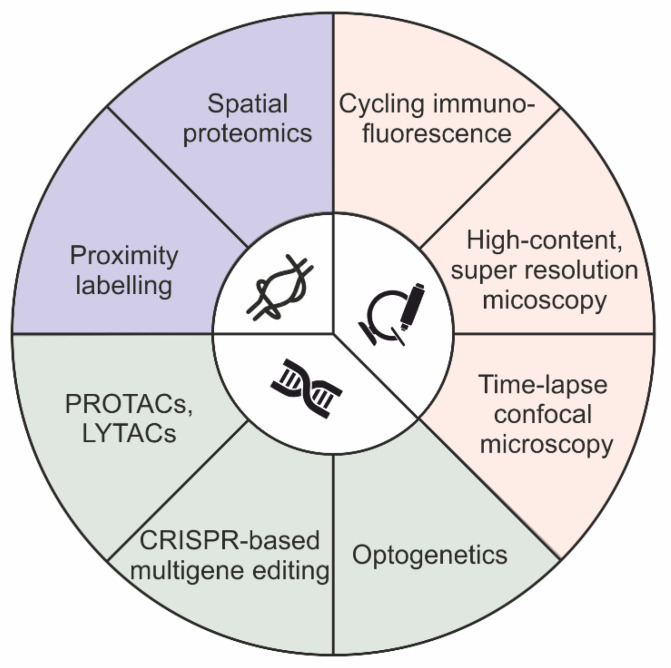
Schematic summary of techniques available for molecular sorting investigations.

There are two main approaches that have historically been used to define distribution of protein cargoes: image-based colocalization analysis and biochemical cell fractionation [24,25]. Colocalization analysis is used to obtain subcellular information about the localization of molecules in both steady-state and stimulated conditions. On the contrary, cell fractionation is limited to the analysis of cargo distribution at the equilibrium. However, while image-based colocalization methods can capture a few molecules on a single sample, it is not biochemical cell fractionation. Notably, during the last ten years, both approaches have been upgraded, thereby expanding their capability to identify cargo distribution, even at high-throughput and in genome-scale genetic screening. In particular, the multiplexing ability of image-based colocalization assays was expanded for the detection of more than 60 distinct channels, using either cycling immunofluorescence or indirect immunofluorescence methods, thereby providing high-throughput imaging of biological samples in both adherent cells and human tissues at the micrometer scale [26,27,28,29]. In addition, the potential of the imaging-based approaches was extended beyond classical gene function studies. Currently, such approaches are implemented in both genome-scale and genome-wide genomic screening, as a result of machine learning and convolutional neural network models [24,30,31,32,33]. In parallel, cell fractionation methods have been combined with mass spectrometry-based proteomics to produce an approach named spatial proteomics [34,35,36,37,38,39,40]. In addition, subcellular fractionation techniques, which do not involve centrifugation, have also been developed, thereby simplifying the identification of protein distribution in organelles [41,42,43]. Notably, such fractionation-based procedures are not compatible with either genome-scale and genome-wide screening, thus limiting their employment for the characterization of a few experimental conditions. Lastly, enzymatic protein biotinylation, in a radius of 10–20 nanometers from the protein of interest, was used as a strategy for interactome mapping of “ad-hoc” proteins, an approach named proximity labeling (e.g., BioID and APEX) [44] (Figure 1).

Before considering the employment of either the image- or biochemical-based method described above for an endosomal sorting investigation, a brief summary of the main features characterizing a protein sorting mechanism is required. The sorting process is a dynamic process with a duration of seconds and is confined to a nanometer-sized space [45,46,47]. Endosomal sorting studies are based on temporal correlation, in which the time variable indicates the directionality of the process and allows us to establish “cause-effect” relationships between distinct components localizing at the same place. Therefore, the employment of widefield-microscopy and quantitative proteomics does not allow a “good coverage” in terms of spatio-temporal resolution. Similarly, super resolution microscopy methods are limited in either frequency at which consecutive images are captured, the fluorophore that could be used, or the toxicity of light illumination [48,49,50]. As a consequence, current studies mainly employ confocal laser scanning microscopy to acquire images with an elevated signal-to-noise ratio and a high frequency, thereby meeting the spatio-temporal requirements for “causality assessment” in molecular sorting. However, to deeply investigate the directionality of the sorting process, time-lapse imaging is not sufficient, as it provides correlation instead of causality in order to connect several cellular processes. In this context, gene function perturbation offers a useful approach to strengthen the “cause-effect” relationship and the directionality of the sorting process under analysis. Classically, gene function perturbations were performed by adding, deleting, and downregulating “ad hoc” genes using a variety of systems, ranging from small interfering RNA to recent CRISPR-mediated genome editing [51,52]. Unfortunately, these approaches are ineffective to address questions concerning both the pleiotropy and redundancy of the sorting machinery. This is due to the onset of compensatory pathways that emerge from the adaptation of the cellular system subsequently to the induction of the “slow acting” genetic perturbation. Nonetheless, recent technologies, such as optogenetic and proteolysis targeting chimera (PROTAC), are able to cope with such issues. While optogenetics allows light-dependent control of protein function with elevated spatio-temporal resolution (micrometer/millisecond), PROTAC involves drug-induced targeted protein degradation by redirecting the ubiquitin–proteasome system [53,54,55,56].

Optogenetic systems are built upon proteins that undergo conformational changes in response to light stimulation at specific wavelengths. Several optogenetic systems are available for researchers, which can be classified based on the number of subunits that are required for functionating, from monomer to multimer [56]. Optogenetic technology is currently employed in a variety of cell biology disciplines, ranging from cell signaling to gene regulation and phase separation to membrane trafficking. In particular, the employment of optogenetic technology in membrane trafficking is well validated in the clusterization of both intracellular membrane cargoes and endocytic regulators [57,58].

PROTACs are small, heterobifunctional molecules composed of two active domains and a linker. PROTAC mediates the formation of the ternary complex (protein target–PROTAC–E3 ligase) by bringing a specific E3 ligase into close proximity of the defined target protein, leading to ubiquitination and degradation of the targeted protein. The protein of interest is expressed as a fusion with a tag protein and subsequent PROTAC treatment allows rapid, reversible and dose-dependent degradation of the fusion peptide [59]. Unfortunately, the PROTAC approach is ineffective for proteins that contain cytosolic domains to which ligands can bind (e.g., extracellular and membrane-associated proteins). In this context, the development of a method using lysosome-targeting chimeras (LYTACs) was recently reported, which allows degradation of membrane proteins by fusing a small molecule or antibody to chemically synthesized glycopeptide ligands that direct the ternary complex to lysosomes via the cation-independent mannose 6-phosphate receptor (CI-M6PR) [60].

## 3. Pathways for Molecular Sorting

**Figure 2:**
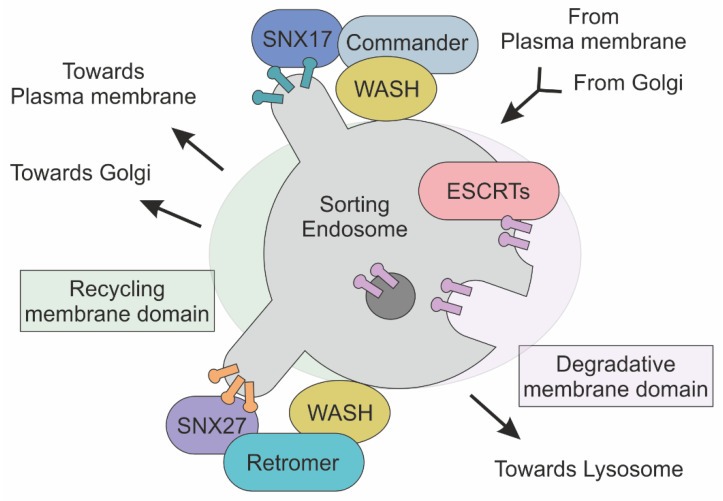
Schematic representation of Commander, ESCRT and Retromer complexes at the sorting endosome. Transmembrane cargoes are directed to plasma membrane, Golgi and Lysosome are accumulated in endosome. In this membrane-bound compartment, the Commander complex, together with the SNX and WASH complexes, mediates the sorting of cargoes to the plasma membrane. In parallel, ESCRT complexes recognize and direct ubiquitylated protein towards the lysosome for degradation. The Retromer complex, in association with the SNX and WASH complexes, defines transport towards both Golgi and the plasma membrane through the association of different SNX proteins.

Early endosomes are crucial sorting platforms for membrane cargoes. Not surprisingly, endosomal sorting dysfunctions are the leading cause of human diseases, including neurodegenerative diseases. Four distinct pathways are summarized below, providing a brief comparative description of the main functional interactions (Figure 2).

### 3.1. Retromer

Retromer was discovered in 1998 during experiments on the characterization of VPS10 receptor recycling in yeast, from the endosome to the trans-Golgi network [61]. Shortly thereafter, Retromer homologue was identified in mammals [62]. Retromer is a heteropentameric complex involved in the recognition of transmembrane proteins at the early endosome. Retromer is involved in the delivery of endocytosed cargoes to both the trans-Golgi network and cell surface [61]. Retromer complex consists of two distinct functional modules: a dimer formed by Vps5p and Vps17 proteins and a trimer composed by Vps35p, Vps29p, and Vps26p (two paralogues, VPS26A and VPS26B, are expressed in humans) [63,64]. Both of these are necessary for both Retromer formation and sorting activity, as endosomal enlargement and protein mistargeting were observed by knocking out Retromer subunits [65]. Retromer subunits recognize two distinct signals localizing at the endosome membrane surface: phosphatidylinositol-3-phosphate (PtdIns(3)P), a phosphoinositide-derived lipid, and hydrophobic signal peptides encoded on the cytoplasmic tails of transmembrane protein cargoes (e.g., sorting motifs or bipartite sorting motifs) [66,67]. The dimeric subunit binds the endosomal-enriched lipid PtdIns(3)P, whereas sorting motifs such as e NPXY or YXXØ are recognized by the Vps35p, Vps29p Vps26p trimeric complex [68]. Once recruited and assembled, the Retromer complex guides creation of filamentous actin (F-actin)-enriched domains, through association with the Wiskott–Aldrich syndrome and SCAR homologue (WASH) complex [69,70]. At the molecular level, the WASH complex is composed of WASH1, FAM21, SWIP (Strumpellin and WASH-interacting protein), Strumpellin, and CCDC53 (coiled coil domain containing protein 53) [71]. FAM21 binds to Vps35, the Retromer subunit, thereby connecting the actin machinery to the molecular sorting apparatus. In particular, the WASH complex promotes the creation of a branched actin network on endosome-enriched cargo domains, a process that strictly depends on the nucleation-promoting factor (NPF) roles of the WASH complex [72]. The mechanical support provided by actin is a key step for the tubulation of membrane and the subsequent release of the endocytic vesicle [73]. In line with this view, inactivation of the WASH complex results in impaired endosomal and lysosomal sorting [74].

The interaction between Retromer and WASH complexes is mediated by Vps35p, the trimeric complex subunit. Notably, the Vps35p scaffolding function is critical for the efficacy of protein sorting at the endosome. This is demonstrated by the key role of Vps35p mutations found in several human diseases such as cancer, Alzheimer’s disease, and Parkinson’s disease [64,75,76]. In addition, loss of Vps35p gene function results in early embryonic lethality, while hemizygosity is exhibited in earlier-onset of Alzheimer’s disease-like phenotypes [75,77]. Neurological defects induced by Vps35 loss are connected with its ability to recruit the WASH complex. Accordingly, Vps35p D620N mutation impairs WASH complex recruitment to the endosome, leading to a reduced actin polymerization and resulting in protein missorting [78].

### 3.2. Commander

In mammalian cells, the recycling function is also executed by the Commander complex. Specifically, Commander is involved in the recycling of cargo from endosome to plasma membrane [79]. Commander is a multiprotein complex composed of two distinct functional modules: Retriever and CCC. Retriever is structurally related to the trimeric Retromer subunit. Retromer and Retriever share the Vps29 protein for coordination and complex assembly. In addition, VPS26C (i.e., DSCR3) and C16orf62 (i.e., Vps35L) share structural homologies with Vps26 and Vps35—the “core” components of Retromer complex [80]. An intriguing aspect of cargo sorting machinery evolution from yeast to mammals is the replacement of a dimeric complex (i.e., Vps5p and Vps17) with a single protein member of the Sorting nexin protein family [81]. SNXs are isoforms of a large protein family encompassing 33 distinct members [82]. Members of the SNX protein family are characterized by the presence of a Phox homology (PX) domain, which mediates phosphoinositide binding [83,84]. In addition, SNXs participate in both membrane deformation and cargo recognition, a role that is frequently associated with the presence of the Bin/Amphiphysin/Rvs (BAR) domain. [85]. As an example, SNX17 was found to be essential in the mediation of binding and subsequent Retriever-mediated sorting of a variety of transmembrane receptors (over 220), including Notch2, integrin α5β1, LRP1, APP, JAG1 and VLDLR as well as SCL family members [71,86,87]. The role of SNXs as adapter proteins, connecting both receptors and lipid membranes to the sorting apparatus, is confirmed by SNX27, a rare SNX family member that lacks the BAR domain [87]. SNX27 controls the membrane localization of over 100 receptors including GLUT1, ATP7A and STEAP3 [88]. Notably, SNX27 depletion in mice results in protein mis-sorting into the lysosomal degradation pathway, while its upregulation enhances synaptic plasticity and is associated with neuroinflammation after spinal cord injury in mice [89].

Commander interaction with the cargo selection module, Retriever, and the actin polymerization unit, WASH, requires the CCC multiprotein complex. The CCC complex comprises two main subunits: the first subunit includes a coiled-coil domain containing proteins such as CCDC22 and CCDC93, whereas the second comprises any of the 10 distinct members that compose the COMMD protein family (i.e., COMMD1-10) [90]. In parallel, CCC and Retriever are closely linked as they share a common subunit (VPS35L) [79]. The evolutionary conserved interactions between both FAM21, the WASH complex subunit, and CCDC22/93 molecules trigger CCC complex recruitment to the endosome [79]. Significantly, CCC deficiency causes impaired recycling of LDL, ATP7A, and Notch receptors [71,91]). In addition, mutations in CCDC22 molecular scaffolds are associated with X-linked recessive intellectual disability (Table 1) [92].

### 3.3. ESCPE-1

Retrieving and recycling trans-membrane cargo proteins on the cytosolic-facing surface of endosomes is controlled by the ‘Endosomal SNX-BAR sorting complex for promoting exit 1’ (ESCPE-1). This evolutionary conserved coat complex couples the recognition of sorting motifs to the BAR domain-mediated biogenesis of cargo-enriched tubulo-vesicular transport carriers. ESCPE-1 consists of heterodimeric combinations of either SNX5 or SNX6 dimerized to either SNX1 or SNX2, and does not require the Retromer trimeric complex (i.e., VPS26:VPS35:VPS29) for its proper functioning [93]. Notably, SNX1, SNX2, SNX5, and SNX6 are also part of the molecular machinery employed by the Retromer for both endosome-to-plasma membrane recycling and endosome-to-TGN retrieval [94,95]. How such differential regulation is achieved is still unclear. However, recent reports suggest that the recognition of specific sorting motifs by SNXs plays a major role in this process. In Retromer-mediated sorting, recycling cargoes are recognized by the trimeric complex, whereas in ESCPE-1, SNXs control the endosomal recycling and retrograde transport of the CI-MPR, as well as the recycling of the IGF1R, by interacting with receptor sorting motifs [96,97]). An intriguing aspect of the ESCPE-1 complex is related to its association with Retromer. In particular, the SNX27-Retromer complex was found to associate with the SNX1 and SNX2 subunits of ESCPE-1. Such functional interaction relies on SNX27-mediated binding of the disordered amino-termini of the SNX1/2 subunits. This event ensures cargo protein retrieval from lysosomal degradation by SNX27-Retromer into ESCPE-1 tubules [88,98,99].

### 3.4. ESCRTs

Early endosomes are characterized by the concomitant presence of multiple sorting pathways. While Commander and Retromer mediate transport to plasma membrane and/or Golgi membranes, the endosomal sorting complex required for transport (ESCRTs) is critical for membrane remodeling, a function that is associated with the formation of multivesicular bodies and protein degradation. ESCRTs are multiprotein complexes that recognize ubiquitinylated cargo as a sorting signal, and by directing them to the lysosome, allow the formation of intraluminal vesicles [100]. Four macromolecular complexes belong to ESCRT family: ESCRT0, ESCRTI, ESCRTII, and ESCRTIII, based on their appearance on the endosomal membrane [101]. ESCRT0 is composed of Vps27/HRS (HGF-regulated tyrosine kinase substrate) and Hse1/STAM (signal transducing adaptor molecule), which mediate interaction with both the endosomal PtdIns(3)P and ubiquitinated moieties of endocytosed receptors [102]. Ubiquitinated transmembrane proteins are clustered by ESCRT0, which binds ubiquitin with low affinity. Notably, ESCRT0 displays a cooperative multivalent binding, as its avidity increases as a function of recognized ubiquitin moieties [103]. Recruitment of ESCRTI and ESCRTII to ESCRT0 increases ubiquitinated cargo recognition, and both cooperate for invagination of endosomal membrane [104,105]. Lastly, recruitment of ESCRTIII induces membrane budding and ubiquitin release [106]. Notably, ESCRTIII creates the concentric spirals required for constriction of newborn vesicles neck, a process that ends after Vps4-mediated ESCRTIII disassembly and the subsequent scission/release of the vesicle [107]. Accordingly, ESCRTIII mutant cells fail to downregulate and degrade cell surface-signaling receptors (Notch, EGFR and many others), resulting in dysfunctional signaling [108]. In the brain, mutations in the endosomal ESCRTIII-complex subunit CHMP2B result in frontotemporal dementia disease [109,110]. ESCRT machinery has been implicated in the biogenesis of exosome, a group of extracellular vesicles that are originated by invaginations of lipid membranes inside endosomes [111]. In particular, the ubiquitin-binding protein ALIX mediates the budding of intraluminal vesicles by interacting with ESCRTIII. Accordingly, depletion of CHMP4, an ESCRTIII subunit, reduces exosome production [112]. Notably, ESCRT-dependent exosome production is promoted by the Syntenin protein, which interacts with transmembrane receptors (i.e., Syndecan) and ALIX to increase receptor clusters and the generation of exosomes [112,113,114]. Among the sorting signals required for exosome production, the small integral membrane protein of the lysosome/late endosome (SIMPLE) is one of the most interesting [115]. Mutations in SIMPLE are linked with decreased exosome biogenesis, while its overexpression causes an hyperproduction of extracellular vesicles. Biochemical analysis revealed the key role of TSG101 and Nedd4, two proteins involved in ubiquitin recognition. In particular, TSG101 specifically recognizes P(S/T)AP aminoacidic sorting signals, whereas proteins harboring PPXY motifs are detected by Nedd4. Remarkably, by recognizing the ubiquitin moieties, Nedd4 is able to sort cargoes into exosomes [116,117].

### 3.5. Lipid Rafts

Not all molecular sorting mechanisms are initiated by large molecular complexes such as ESCRT and Retromer. Over 30 years ago (i.e., 1988), it was observed that some portions of the plasma membrane, called lipid rafts (LR), act as membrane organizers by controlling both composition and function of biological membranes [118]. LR are enriched in ceramide, cholesterol, sphingolipids, and GPI-anchored proteins. Further investigations individuate that ceramide molecules induce spontaneous membrane invagination and are able to cluster receptors by reducing lateral diffusion of membrane proteins [119,120]. Similarly, endocytic LRs are molecularly heterogenous, thereby conferring both morphological and functional differences. As an example, early endosomes and recycling endosomes are enriched in cholesterol, sphingomyelin, and phosphatidylserine and caveolin. Conversely, these same components are largely depleted from late endosomes [121,122,123,124,125].

Although it is not entirely clear whether the sorting process is actively mediated by lipid rafts, the partial depletion of lipid raft/ caveolae components results in receptor mis-sorting [118,126,127,128]. The LR-mediated pathway is parallel to the degradation and recycling pathway, due to the fact that lipid rafts sort different cargoes and do not interact with Retromer/Retriever or ESCRT [129]. In particular, LRs are necessary for tetraspanin-mediated sorting. Tetraspanins are characterized by four transmembrane domains containing conserved polar residues, a small extracellular loop (SEL), a large extracellular loop (LEL), and short cytoplasmic tails. The C-terminal cytoplasmic tails of tetraspanin CD63 is crucial for proper targeting to intracellular compartments, as it contains a tyrosine-based sorting signal. Notably, mutations in this motif caused CD63 to lose its intracellular localization and traffic to the cell surface [130,131].

**Table 1:** List of sorting genes associated with neurological disorders.

Gene	Inheritance	Disease	References
CCDC22	X-linked recessive	Ritscher–Schinzel syndrome 2; intellectual disability	[92]
Strumpellin	Autosomal Recessive	Ritscher–Schinzel syndrome 1	[132]
	Autosomal Recessive	Spastic paraplegia 8, autosomal dominant	[133]
C16orf62	Autosomal Recessive	Ritscher–Schinzel syndrome 3	[134]
VPS26C		Down syndrome	[135]
VPS35	Autosomal dominant	Parkinson disease 17	[136]
RAB7	Autosomal dominant	Charcot–Marie–Tooth disease, type 2B	[137]
SWIP	Autosomal dominant	Mental retardation, autosomal recessive 43	[138]
RAB11B	Autosomal dominant	Neurodevelopmental disorder with ataxic gait, absent speech, and decreased cortical white matter	[139]
CHMP2B	Autosomal dominant	Frontotemporal dementia and/or amyotrophic lateral sclerosis 7	[110,140,141]
CHMP4B	Autosomal dominant	Cataract 31, multiple types	[142]

## 4. Timing in Molecular Sorting

**Figure 3:**
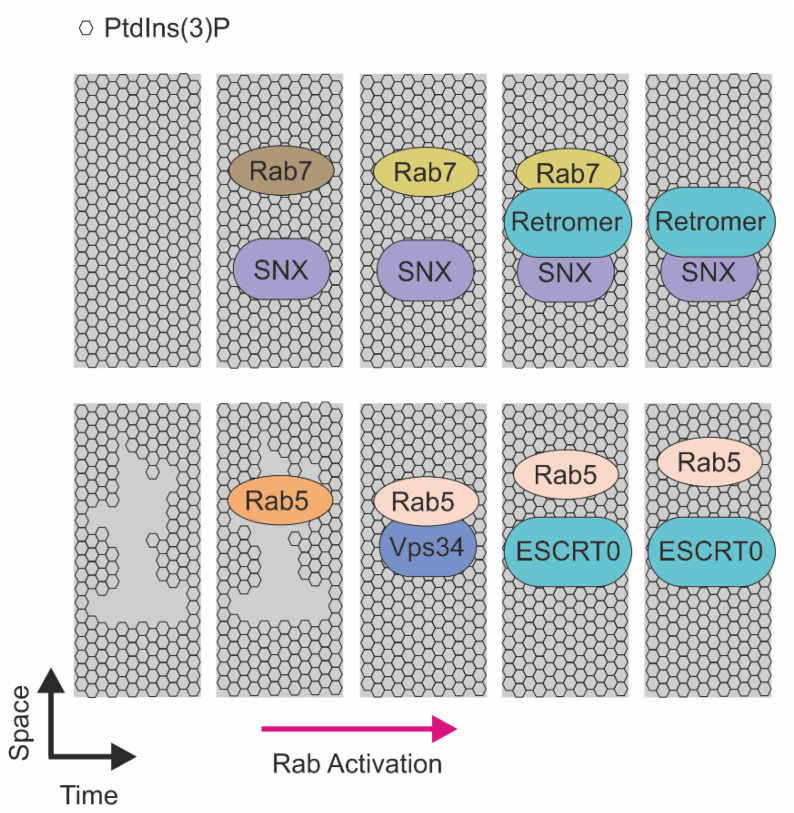
Schematic representation of the RABs-mediated recruitment of sorting machineries on the endosomal membrane. (Top) Recruitment of sorting machineries in function of RAB7. SNX and RAB7 recruitment on the membrane enriched in PtdIns(3)P that, in a sequential manner, mediate endosomal recycling. Activated RAB5 recruits Vps34 and induces PtdIns(3)P production, this event allows ESCRT0 to recognize ubiquitinated proteins on endosome membrane enriched in PtdIns(3)P and direct to lysosome for degradation.

Experimental attempts to reveal the mechanisms of molecular sorting are directed by functional interactions controlling both protein and lipid dynamics on early endosomes (Figure 3). In this context, seminal studies provide the evidence that Retromer machinery biochemically interacts with Rab7, a protein involved in the progression of early endosome to late endosome. Rab7 is a small GTPase that cycles between active and inactive forms to control formation, transport, and delivery of membrane cargoes by interacting with molecular motors such as dynein–dynactin complexes. The switching of Rab7 from an inactive to an active form is proposed as a mechanism for recruitment of the Vps29 subunit to early endosome, and consequently to promote localization of Retromer to endocytic membranes. The pivotal function of Rab7 on sorting endosomes is highlighted by the evidence that depletion of such a small GTPase causes Vps29 mislocalization and major defects in Retromer function [143,144]. In addition, Rab7 colocalizes with Vps29 during the outgrowth of the recycling vesicle in a segregated portion of the endosome corresponding to the recycling tubule [143]. Once detached, vesicles are negative for Rab7 and positive for Vps29. Stability of the Retromer complex to the newborn vesicle strictly depends on SNX proteins, as consistent SNXs depletion results in a block of the sorting process [81]. Similarly, reduction of PtdIns(3)P, the SNXs regulator, results in a similar phenotype [143,145].

The molecular link between PtdIns(3)P and small GTPases is strengthened by the evidence that Rab5 is a master regulator of the PI3K enzymes responsible for phosphoinositide generation on endosomal membranes. In particular, Rab5 activation promotes recruitment of Vps34, the class III PI3K, that, in turn, controls the generation of PtdIns(3)P on the sorting endosome. The presence of concomitant active Rab5 and PtdIns(3)P signals promotes the recruitment of several effectors, including Hrs—the ESCRT0 subunit. Notably, Rab5 localizes with Hrs, while it strictly depends on PtdIns(3)P. Accordingly, depletion of this lipid induces cytoplasmic localization of Hrs [146,147]. In addition, for Rab5 and Rab7, another Rab protein is involved receptor sorting. In particular, Rab11 was found to direct Transferrin receptor sorting at the early endosome through regulation of the PtdIns(3)P level [148,149].

Rab11 activation initiates on endosome membranes enriched in PtdIns(3)P, and reduction of such lipids resulted in a decreased number of released vesicles and an increased residence of time of Rab11 in the PtdIns(3)P positive compartment. Notably, a PtdIns(3)P burst was detected few seconds before the detachment of the sorting vesicle and is concomitant with the increased activation of Rab11. These results demonstrate the important role of PtdIns(3)P in endocytic sorting of cargoes, and highlight how Rab11 activity may be used as a metronome to measure the timing of sorting.

## 5. Conclusions

The continuous and rapid assembly of molecular machineries involved in the molecular sorting processes require a precise timeline of events. Sorting processes are evolutionarily conserved among species and their dysfunction is correlated with several neurodegenerative diseases. Advances in proteomics, microscopy, and genetic engineering approaches will provide a deeper understanding of the sequence of events controlling sorting pathways. Although processes of molecular sorting are currently under investigation, there are still many doubts about their sequential recruitment. Novel studies focused on the identification of molecules that may function as a “metronome” in the control of sorting complex recruitment will provide great value in the field of membrane trafficking.
